# Atlanta Academy of Medicine

**Published:** 1878-02

**Authors:** J. G. Westmoreland

**Affiliations:** Reporter


### Reports of Societies.

### ATLANTA ACADEMY OF MEDICINE.

J. G. WESTMORELAND, M.D., Reporter.

Atlanta, Ga., January 14, 1878.

Dr. J. F. Alexander, President, in the chair.

Dr. A. W. Calhoun read an Essay for the month on "The Influence of School-life on Eye-sight."

Dr. Baird asked why reading, while in the recumbent position, is more injurious than the upright posture?

Dr. Calhoun replied that the horizontal position tended to increase of blood about the head and eyes, which, with the congestion consequent upon the act of reading, proves injurious by leading to softening of the tissues, and finally, if constantly practiced, to partial paralysis of the retina.

Dr. Baird thought the position of the book, causing the light to strike it at an unfavorable angle, is, perhaps, the main source of trouble from this mode of reading.

Dr. Calhoun accepted this as one of the sources of trouble.

Dr. Taliaferro reported the case of a lady thirty-five years old, who had fallen from the door steps upon the buttox, producing considerable concussion. This was succeeded by persistent stricture of the bowels. Epsom salt was administered before he was called to the case, and the contents of the lower bowels had been evacuated. After this, castor oil was given in large quantity, without other effect than its rejection from the stomach. Injection of water with a Davidson syringe was then had to the full extent that was possible. Not more than a quart or two of water, however, could be forced into the colon. The patient did not seem greatly disturbed with pain or constitutional depression, and it was thought advisable to give an active cathartic, and croton oil to the amount of four or five drops was administered without purging effect. Chloroform was inhaled during the injection of water, with the hope of making a more successful filling of the bowel, but without realizing any advantage from it. Opiates were occasionally given, and with the effect of keeping the patient comparatively free from pain. Calomel—saccharated and plain—was given at several times during the treatment. About a week from the accident of the fall, and supposed consequent obsruction, the passage of fecal matter baving ceased for at least four or five days, as a last resort, forcible injection of water was repeated, without the ability to introduce more than a few pints. The patient gradually declined, and died about the tenth day of the obstruction.

Considerable discussion was had by members in regard to the location and nature of the obstruction in this case.

Dr. Taliaferro himself was inclined to the opinion that the cœcum was the point of difficulty, from the existence in that region of occasional pain and a rather firm tumefaction. At the same time, the small quantity of water retained in the bowel would seem to favor the idea of stricture lower down in the large bowel, as the small amount of water that could be injected was not sufficient to fill the whole colon. It had been ascertained by exploration of the bowel as high as the sigmoid flexure of the colon, by introduction of the hand, that no scybala or other cause of obstruction existed in that part. While he attributed the obstruction to the fall, he could not well understand why it affected the bowels, or what manner of derangement of the bowel was produced by it, leading to such uncontrollable obstruction.

Other members agreed with him in supposing vertical concussion from the fall led to the difficulty, but were also unable to give a satisfactory explanation of the effect upon the bowel.

Dr. Rauschenberg thought the introduction of a flexible tube through the sigmoid flexure might have proved more successful in forcing a larger quantity of water into the colon.

Dr. J. M. Johnson favored the flexible tube, and alluded to the successful treatment of other cases by this means.

Dr. J. T. Johnson could not understand how the tube could possibly facilitate the passing of water into the colon, as the pressure from the pump would certainly drive the water through any part that the tube would pass.

Dr. J. G. Westmoreland thought the obstruction existed not far above the sigmoid flexure, and was forced to this opinion from the small quantity of water that could be injected. He was of opinion that such cases should have the abdomen opened when all other means had failed to remove the obstruction. When asked his opinion of the nature of the obstruction, he expressed his belief in the possibility of invagination of the colon in the neighborhood of the sigmoid flexure, probably, from the sudden, violent vertical concussion in the fall.

Dr. J. G. Westmoreland reported improvement in the case of abscess of the inguinal canal, operated on a few weeks since. The discharge has nearly ceased, and the improvement in strength and general appearance is marked.

Adjourned.

---

Atlanta, Ga., January 21, 1878,

Dr. J. F. Alexander, President, in the chair.

Dr. A. W. Calhoun presented the specimen of a melanotic cancer, in the person of a young man twenty-four years old. The history, as given, is that a point of disease was discovered through the pupil some three or four years since. The disease continued, and when he was presented the whole orbit was filled with the cancerous growth; so much so that the extirpation was with difficulty effected. It was found that the optic nerve was the subject of the same melanotic cancerous degeneration, even to the optic foramen, where it was clipped. The prognosis is unfavorable, therefore, as the cancerous condition doubtless exists within the cranium. The case seems to be doing well, however—now four days since the operation—and but for the extension along the optic nerve would give hope of recovery. Again, the history of cases of this disease, commencing, as this did, in the retina, leads also to unfavorable prognosis.

Dr. Alexander, the President, asked of members whether they had met with scarlet fever in their practice. He had met with a case, the patient of another physician, the disease having been contracted in Florida. The question was raised in connection with this case, as to the nature of the eruption in scarlet fever, compared with that found in diphtheria. Dr. Alexander thought the characteristic symptom of scarlatina, and one almost amounting to a pathognomonic, is that of discoloration of the reddened surface by passing the hand over it. He, however, with some other members, had found the eruption to exhibit roughness to the touch, resembling fine sand under the fingers.

Adjourned.

---

ATLANTA, Ga., February 4, 1878.

Dr. J. F. Alexander, President, in the chair.

Dr. A. W. Calhoun reported a case of epithelial cancer of the cornea, in a lady some fifty years old. The case required enucleation, which operation was made two days ago. The case is peculiar, in that this kind of disease rarely commences in the cornea. At first simple ulceration was supposed to be the only trouble, but more thorough investigation proved its malignant character. The case has been under treatment about one month, and showed the first symptom of disease six months ago.

Dr. Calhoun also referred to the production of cancer by cauterizing the conjunctiva with nitric acid and nitrate of silver for pterygium. Some months since the opinion of such result was expressed by Dr. C., and attacked by members who were of the opinion that cancerous disease is not the result of the cauterization, but occurs on account of the germ or cancer cell existing previously. He had a case recently which, beyond doubt, occurred from the same application of the nitrate improperly in a case that would otherwise have terminated favorably.

Dr. Baird reported an unusual case of dislocation of the inferior maxilla from a spasmodic contraction of the muscles of the face and neck. The patient had been the subject of paralysis of the right side from cerebral embolism, but had recovered for some months before this attack of convulsive or spasmodic action about the face, which resulted in the dislocation.

Dr. Baird called up the subject of inflammation of the tonsils, and mentioned a case with which he had met recently. He has found great difficulty in getting the mouth open sufficiently to inspect the part inflamed. He desired the experience of members as respects this difficulty.

Others had similar experience in this particular. Dr. Calhoun found the laryngiscope useful in making such examination, and advises incisions made in the tonsils before and after suppuration has been established.

Adjourned.

---

ATLANTA, GA., February 11, 1878.

Dr. J. F. Alexander, President, in the chair.

Dr. W. F. Westmoreland reported an operation for stone in the bladder, by median cut. The case had been operated on several months ago for stricture of the urethra, by perineal section, from which a clot probably was left and served as a nucleus for the stone. The subject, fifty years old, and now second day from the operation, is doing exceedingly well. This is the first operation by the median section he ever made, and it was performed by cutting through the perinæum, commencing on the median line an inch from the anus, in the direction of the membranous portion of the urethra, into which a director had been introduced. The cut was made through the membranous portion alone, without cutting the bulbous portion or neck of the bladder. After dilating the neck of the bladder with the finger, a small pair of forceps was introduced, and a stone somewhat kidney-shaped, an inch long and half an inch in diameter, was extracted.

Adjourned.

